# Unveiling G-protein coupled receptors as potential targets for ovarian cancer nanomedicines: from RNA sequencing data analysis to in vitro validation

**DOI:** 10.1186/s13048-024-01479-0

**Published:** 2024-07-27

**Authors:** Riya Khetan, Preethi Eldi, Noor A. Lokman, Carmela Ricciardelli, Martin K. Oehler, Anton Blencowe, Sanjay Garg, Katherine Pillman, Hugo Albrecht

**Affiliations:** 1 Centre of Pharmaceutical Innovation, UniSA Clinical and Health Sciences, University of South Australia, Adelaide, South Australia 5000 Australia; 2 UniSA Clinical and Health Sciences, University of South Australia, Adelaide, South Australia 5000 Australia; 3 Discipline of Obstetrics and Gynaecology, Adelaide Medical School, Robinson Research Institute, University of Adelaide, Adelaide, South Australia 5000 Australia; 4 Department of Gynaecological Oncology, Royal Adelaide Hospital, Adelaide, South Australia 5000 Australia; 5 Applied Chemistry and Translational Biomaterials Group, Centre of Pharmaceutical Innovation, UniSA Clinical and Health Sciences, University of South Australia, Adelaide, South Australia 5000 Australia; 6 Centre for Cancer Biology, UniSA Clinical and Health Sciences, University of South Australia, Adelaide, South Australia 5000 Australia

**Keywords:** Ovarian cancer, GPCR, Nanomedicine, Personalised medicine, Drug delivery, RNA-seq

## Abstract

Genetic heterogeneity in ovarian cancer indicates the need for personalised treatment approaches. Currently, very few G-protein coupled receptors (GPCRs) have been investigated for active targeting with nanomedicines such as antibody-conjugated drugs and drug-loaded nanoparticles, highlighting a neglected potential to develop personalised treatment. To address the genetic heterogeneity of ovarian cancer, a future personalised approach could include the identification of unique GPCRs expressed in cancer biopsies, matched with personalised GPCR-targeted nanomedicines, for the delivery of lethal drugs to tumour tissue before, during and after surgery. Here we report on the systematic analysis of public ribonucleic acid-sequencing (RNA-seq) gene expression data, which led to prioritisation of 13 GPCRs as candidates with frequent overexpression in ovarian cancer tissues. Subsequently, primary ovarian cancer cells derived from ascites and ovarian cancer cell lines were used to confirm frequent gene expression for the selected GPCRs. However, the expression levels showed high variability within our selection of samples, therefore, supporting and emphasising the need for the future development of case-to-case personalised targeting approaches.

**Supplementary Information:**

The online version contains supplementary material available at 10.1186/s13048-024-01479-0.

## Introduction

Ovarian cancer significantly contributes to the global cancer burden and mortality, with more than 300,000 cases per year and nearly as many deaths [1]. Due to the lack of screening strategies and non-specific symptoms, it is mostly diagnosed at an advanced stage, resulting in a 5-year survival rate of 26–42% [2, 3]. Despite the advancement in frontline treatment (platinum-based chemotherapy and debulking surgery [4]) and maintenance therapy (e.g., poly-adenosine diphosphate- ribose) polymerase inhibitors [5]), the genetic heterogeneity and aggressive nature of ovarian cancer often leads to poor treatment response, drug resistance and relapse [6, 7]. Moreover, the early and widespread intra-abdominal metastasis of ovarian cancer affects the treatment efficacy and is associated with significant morbidity [8]. These drawbacks and the unique genetic makeup of ovarian cancers warrant the discovery of new biomarkers in combination with personalised targeted precision nanomedicines to improve patient outcomes. Such approaches might be further enhanced by using the intra-peritoneal delivery route for nanomedicines, leveraging the abdominal cavity’s proximity to ovarian tumours, and enhancing the efficacy of therapeutic agents by ensuring higher local drug concentrations at the tumour site while minimising systemic exposure and side effects.

However, in the realm of ovarian cancer, very limited precision medicines have made their way into clinical applications, particularly for targeted nanoparticles. In contrast, some notable successes have been achieved for other cancer types with antibody drug conjugates, leading to improved treatments based on receptor targeting [9, 10]. Mirvetuximab soravtansine is a recently developed antibody drug conjugate for ovarian cancer treatment consisting of a humanised monoclonal folate receptor alpha (FR-α) antibody, which is linked *via* a cleavable disulphide bond to maytansinoid DM4, a synthetic derivative of the highly potent cytotoxic agent maytansine. Consistent antitumour activity has been demonstrated in patients with high FR-α expression, along with a favourable safety and tolerability profile [11]. Another example demonstrated the development of multiplexed magnetic nanoparticle-antibody conjugates to quantify the presence of three biomarkers: carbohydrate antigen 125 (CA-125, MUC16), b2-microglobulin (β2-M) and apolipoprotein A-I (APOA-I) [12]. This enabled the detection of early-stage ovarian cancer by achieving higher sensitivity (94%) and specificity (98%) [12]. While nanoparticle-based strategies show promise in ovarian cancer, their application has predominantly been focused on diagnostics [13]. Despite extensive research and development efforts, approved ovarian cancer nanomedicines, using active cancer cell targeting mechanisms remain elusive [14].

Recent advancements have triggered interest in the potential use of GPCRs for diagnostic purposes and the development of radiopharmaceuticals. GPCRs with their signature seven-transmembrane domain structure form the most versatile and largest cell surface receptor family with approximately 800 members [15], and they are easily accessible from the extracellular space. The dysregulation of GPCRs has been frequently implicated in cancer progression, making these receptors attractive targets for therapeutic intervention with ligand-decorated drug carriers [16]. Although these receptors have not been used as theranostic targets in the context of ovarian cancer, they are most promising for the future development of precision therapy using nanomedicines.

The concept of targeted drug delivery has gained attraction in recent years to enhance the efficacy of anticancer agents while minimising off-target effects. By studying the overexpression of GPCRs in ovarian cancer, we propose a paradigm shift in drug delivery strategies. This will include the use of GPCRs to specifically target ovarian cancers in a personalised manner using tailor made nanomedicines for future treatment. This approach holds promise for addressing the challenges associated with current front-line treatment, such as systemic toxicity and drug resistance.

The advancement in precision medicines, using specially tailored drugs and treatment options, has been accelerated by publicly available large-scale genetic data. This is of utmost interest in ovarian cancer due to the challenges associated with the highly diverse genetic makeup. Using RNA-seq data as a starting point allows to the rapid, systematic analysis and identification of potential targets, including overexpressed GPCRs. For this study, we used publicly available RNA-seq data to identify overexpressed GPCRs in ovarian cancer tissues. Our study revealed 13 overexpressed GPCRs when comparing cancer tissues to healthy tissues. Furthermore, expression of the 13 GPCRs was investigated in patient-derived ovarian cancer ascites samples and established ovarian cancer cell lines. In summary, we propose to bridge the gap between molecular profiling and clinical translation by identifying overexpressed GPCRs as molecular entry points for targeted drug delivery to advance ovarian cancer treatment, potentially offering maintenance therapies to manage the disease over the long term.

## Materials and methods

### Materials

Ovarian cancer cell line ES-2 (RRID: CVCL_3509) was purchased from Sigma-Aldrich (NSW, Australia). CaOV3 (RRID: CVCL_0201), OVCAR3 (RRID: CVCL_0465) and SKOV3 (RRID: CVCL_0532) cell lines were purchased from American Type Culture Collection (ATCC, Manassas, VA, USA). COV362 (RRID: CVCL_2420) was purchased from European Collection of Authenticated Cell cultures (ECACC, Salisbury, UK). The use of patient-derived ascites samples was approved by the Royal Adelaide Hospital Human Research Ethics Committee (Ethics number HREC/14/RAH/13 and HREC/18/CALHN/811) with patient informed consent. Roswell Park Memorial Institute (RPMI 1640), Dulbecco’s modified eagle’s medium-high glucose (DMEM), fetal bovine serum (FBS), penicillin-streptomycin (10,000 U/mL), Dulbecco’s phosphate buffered saline (DPBS), trypsin-ethylenediaminetetraacetic acid (EDTA), trypan blue solution and the primer sequences for real-time quantitative polymerase chain reaction (RT-qPCR) were purchased from Sigma-Aldrich (NSW, Australia). Advanced RPMI 1640 medium, GlutaMAX™ supplement (100×), and countless cell counting chambers were purchased from Thermo Fisher Scientific (Life Technologies, Australia). RNeasy^®^plus mini kits were purchased from Qiagen (VIC, Australia). Hard shell^®^ 96-Well PCR plates, Microseal ‘B’ PCR plate sealing film, iScript™ cDNA synthesis kit and iTaq™ universal SYBR^®^ green supermix were purchased from Bio-Rad (NSW, Australia). Primary antibodies: C-X-C chemokine receptor type 4 (CXCR4) monoclonal (60042-1-UG) and coagulation factor II thrombin receptor (F2R) polyclonal (26366-1-AP) were purchased from United Bioresearch (Australia). Secondary antibody fluorescein isothiocyanate (FITC) goat anti-mouse IgG (H + L) and FITC goat anti-rabbit IgG (H + L) were purchased from Abclonal via Genesearch (Australia). Cytofix/Cytoperm kit was purchased from BD Biosciences (New Jersey, USA).

### Collection of RNA-seq data and selection of GPCRs

The National Center for Biotechnology Information (NCBI) database was thoroughly searched to identify Gene Expression Omnibus (GEO) datasets with RNA-seq data relevant to ovarian cancer. Six datasets including one healthy ovarian tissue dataset were chosen and the raw data for each GEO dataset was downloaded from the GEO RNA-seq experiments interactive navigator (GREIN) database [17]. Quality control of these data was done by calculating counts per million (CPM) and only choosing genes with CPM values of more than 1 in at least 70% of samples. All samples were normalised according to the trimmed mean of M-values (TMM) method [18] and the normalised data were converted into reads per kilobase per million mapped reads (RPKM) using an R script adapted from the Bioconductor package [19]. A Venn diagram was generated using free software from the Van de Peer laboratory, Ghent University, Belgium (bioinformatics.psb.ugent.be/webtools/Venn/).

### Culture of ovarian cancer cell lines and primary ovarian cancer cells

The cell work was performed in a biosafety cabinet and all cultures were confirmed mycoplasma free using the Mycoalert detection kit (Lonza, Australia). ES-2, OVCAR3 and SKOV3 cell lines were maintained in RPMI medium with 10% (v/v) FBS and 1% (v/v) penicillin-streptomycin. CaOV3 and COV362 cell lines were maintained in DMEM supplemented with 10% (v/v) FBS and 1% (v/v) penicillin-streptomycin. Cells were grown in a humidified atmosphere of 95% air and 5% CO_2_ at 37 °C (Sanyo CO_2_ incubator) until at least 90% cell confluency was reached.

All cell lines except SKOV3 were authenticated on 12th April 2021 using short tandem repeat analysis. This was performed with the Promega GenePrint^®^ 10 system (Griffith University DNA sequencing facility, QLD, Australia). SKOV3 cells were authenticated by the Australian Genome Research Facility (AGRF), South Australia on 14th October 2022.

Table 1 includes the clinical information on the patient ascites samples that were used to isolate the primary ovarian cancer cells. Primary ovarian cancer cells were cultured in advanced RPMI medium supplemented with 10% (v/v) FBS, 1% (v/v) penicillin-streptomycin and 1% (v/v) GlutaMAX™. Cells were maintained at 37 °C with 5% CO_2_ as previously described [20].

**Table 1:** Clinical information on patient-derived samples

No	Age at diagnosis	Diagnosis	Stage (Grade)	Resistant/ Sensitive
A1	81	Recurrent, chemotherapy resistant serous peritoneal carcinoma	*4 (3)*	*R*
A2	80	Primary peritoneal carcinoma	*3c (3)*	*S*
A3	69	Recurrent ovarian carcinoma	*3a (3)*	*R*
A4	57	Recurrent chemotherapy resistant ovarian carcinoma		*R*
A5	68	Serous carcinoma of the ovary	*4 (3)*	*S*
A6	47	Recurrent serous carcinoma of the ovary	*1 C (3)*	*R*
A7	59	Serous ovarian carcinoma	*3 (3)*	*S*
A8	53	Serous carcinoma of the ovary	*2a (3)*	*S*
A9	71	Recurrent chemotherapy-resistant serous carcinoma of the ovary	*3c (3)*	*R*
A10	73	Serous carcinoma of the peritoneum	*4 (3)*	*S*

R: Chemotherapy resistant. S: Chemotherapy sensitive

### Real-time quantitative PCR analyses

Cells were detached using trypsin and followed by centrifugation for RNA isolation with a RNasy^®^plus mini kit. The isolated RNA was used to synthesise cDNA using an iScript™ cDNA synthesis kit, according to manufacturer’s instructions. RNA quality and concentration were analysed using a NanoDrop 1000 spectrophotometer (Thermo Fisher Scientific).

RT-qPCR was performed using iTaq™ Universal SYBR^®^ Green Supermix and was run on a CFX Connect™ Real-Time System (Bio-Rad). Each 15 µL reaction mix contained 5 µL of SYBR^®^ Green Supermix, 2 µL each of forward and reverse primers (10 µM), 2 µL of cDNA and 4 µL of RNase-free water. Detailed information of all primers is given in Tables S1, S3 and Figure S4.

PCR cycling conditions included hot start for 15 min at 95 °C followed by 95 °C for 15 s, 57 °C for 20 s and 72 °C for 20 s (with 45 cycles following 95 °C for 1 min). Cycle threshold (C_t_) values were determined and delta C_t_ (ΔC_t_) values were calculated by using the human succinate dehydrogenase complex flavoprotein subunit A *(SDHA)* housekeeping gene as internal standard. All experiments were run in triplicates.

### Flow cytometry

Cells were harvested using trypsin and resuspended in fluorescence-activated cell sorting (FACS) buffer (PBS + 2% FBS). 1 × 10^6^ cells were stained with live/dead marker (1:3000) in DPBS (20 min at 4 °C). Cells were then washed, fixed and permeabilized using a Cytofix/Cytoperm kit as per the manufacturer’s instructions, prior to incubation with 1:100 dilution of primary antibody in permeabilization buffer (30 min at 4 °C) followed by incubation in 1:100 dilution of secondary antibody in permeabilization buffer (30 min at 4 °C). Cells were finally resuspended in FACS buffer for flow cytometry analysis. Data were acquired using FACSAria™ Fusion (BD Biosciences, New Jersey, USA), and analysed using FlowJo™ version 10 software (BD Biosciences, New Jersey, USA).

## Results and discussion

### Selection of RNA-seq data sets

**Fig. 1:**
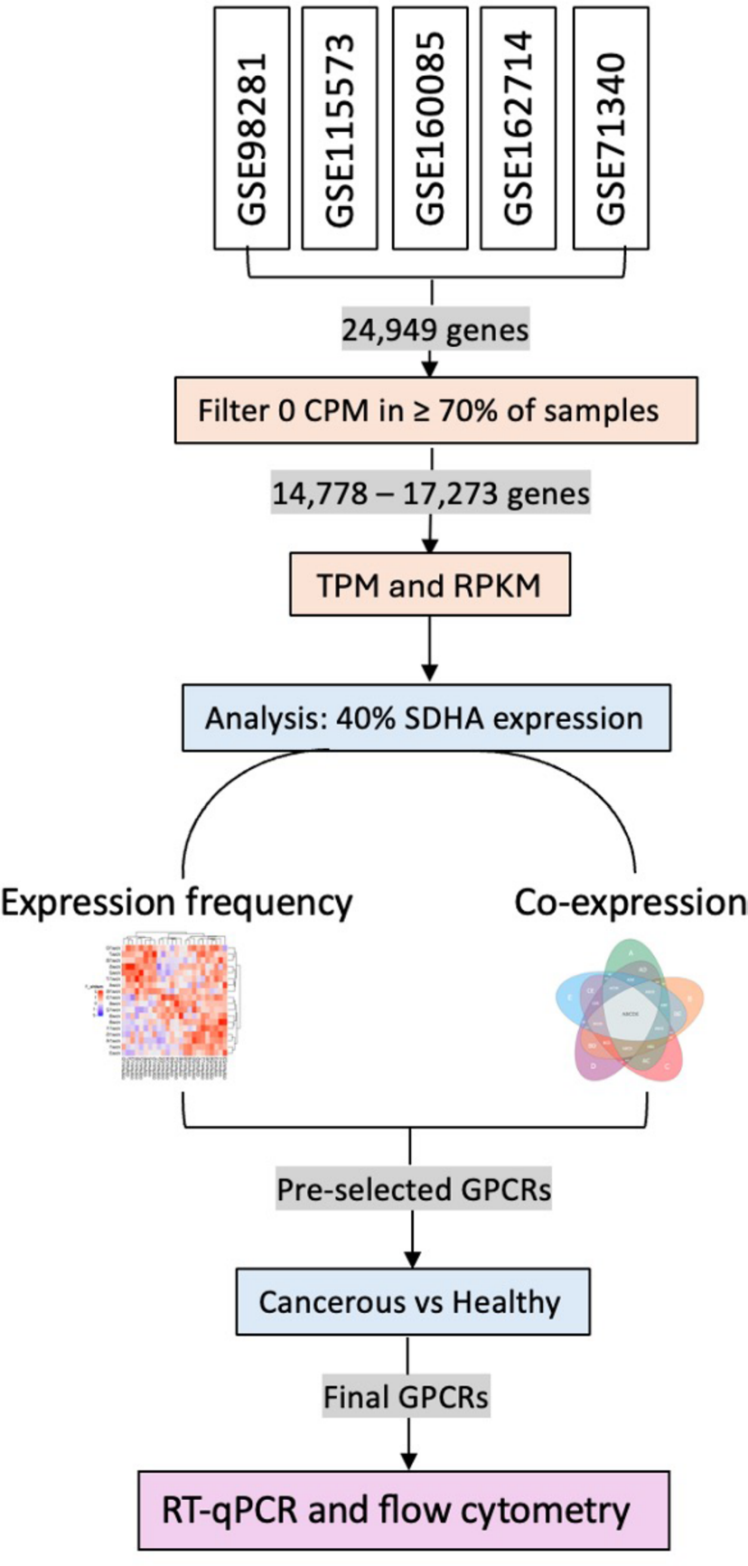
Workflow for the identification of overexpressed GPCRs in ovarian cancer tissues. The following stages were involved: Collection of RNA-seq raw data, removal of genes with no or very low expression based on CPM values, followed by TPM and RPKM quantification. Subsequent analysis was focused on genes with expression levels of at least 40% relative to the *SDHA* housekeeping gene, assessing expression frequencies within datasets and co-expression patterns between datasets (Venn analysis). The pre-selected candidate GPCRs were run through a final selection excluding candidates with high expression in healthy tissue samples. The remaining candidate GPCRs were validated using RT-qPCR and flow cytometry with primary ovarian cancer ascites cells and ovarian cancer cell lines

The initial goal was to systematically identify GPCRs with significant gene expression in ovarian cancer tissues and low expression in healthy ovarian tissues. In the past, public microarray data have been studied for similar purposes [21]. Since RNA-seq is known to be more sensitive and accurate than microarray analysis, especially for low expressed genes [22, 23], we conducted a systematic search of the NCBI GEO database to identify RNA-seq data sets which have been generated from various human high-grade serous ovarian cancer tissues. Only studies with primary ovarian cancer tissues were considered; therefore, experiments with in vivo cell lines and xenograft models were excluded. In addition to this, data sets with higher sample numbers were prioritised. Table 2 summarises five datasets which were selected for our analysis, representing various tumour types and stages, including pre and post chemotherapy, and neoadjuvant chemotherapy (NACT). An additional dataset, which contained healthy tissue samples only (GSE114493) was used for comparison to identify overexpressed GPCRs in ovarian tumour tissues. Raw data from individual samples of the selected datasets were subjected to the workflow as outlined in Fig. 1.

**Table 2:** Public RNA-seq data chosen for the analysis

Data set accession	Sample size	Type of cells (number of samples)
**Cancer samples (all high-grade serous ovarian cancer)**		
GSE162714 [24]	77	Pre-chemo (*n* = 22), Post chemo (*n* = 55)
GSE115573	9	Primary (*n* = 3), Omentum (*n* = 3), Effusion (*n* = 3)
GSE160085 [25]	10	Pre-chemo high grade serous (*n* = 10)
GSE98281 [26]	20	Primary (*n* = 10), Metastatic (*n* = 10)
GSE71340 [27]	35	Pre NACT (*n* = 17), Post NACT (*n* = 18)
**Healthy samples**		
GSE114493 [28]	10	Healthy cells (Fallopian (*n* = 3), Peritoneal (*n* = 3), Ovarian surface (*n* = 3))

### Data processing

Raw count files of the selected GEO datasets were retrieved from the GREIN database [17]. The edgeR Bioconductor package was used to convert the data into CPM [29], and genes with no expression (CPM values equal to zero) in more than 70% of samples within individual GSE datasets were discarded prior to TMM normalisation to adjust the gene expression values across samples [18]. An example boxplot before and after normalisation is shown in Figure S1 for dataset GSE98281.

**Fig. 2:**
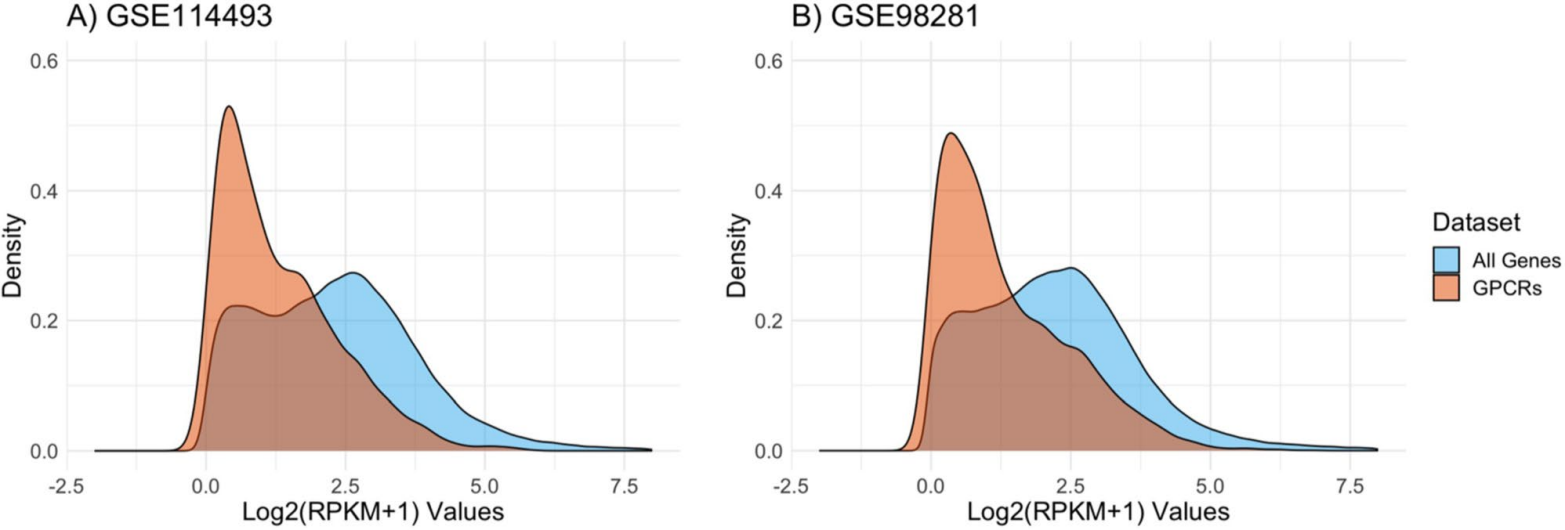
Kernel density estimation to evaluate the distribution of RPKM values across ten healthy samples from GSE114493 (left) and 20 cancerous samples from GSE98281 (right)

The data were transformed into RPKM to compensate for differences of gene length, therefore facilitating comparison of gene expression levels within samples [30]. Kernel density plots were generated to assess the distribution of gene expression levels for all remaining genes versus GPCRs only [31]. Figure 2 shows results for 17,105 genes and 209 GPCRs from a dataset of 10 healthy tissue samples (GSE114493), and 17,273 genes and 211 GPCRs for a dataset of 20 cancer samples (GSE98281). The distributions for an additional four datasets are given in Figure S2. A broad distribution of expression levels was observed for all datasets when considering all genes. In contrast, GPCRs showed relatively narrower curves with a strong bias towards low to moderate expression. A generally low expression is expected for this cell surface receptor family in contrast to many other proteins, which are located in the cytoplasmic space and require higher expression levels to maintain cellular functions​ [16]. To reach effective receptor-mediated drug delivery into cancer cells, the low to moderate receptor expression is expected to be leveraged by targeted nanoparticles carrying large drug loads.

### Pre-selection of target receptors

**Fig. 3:**
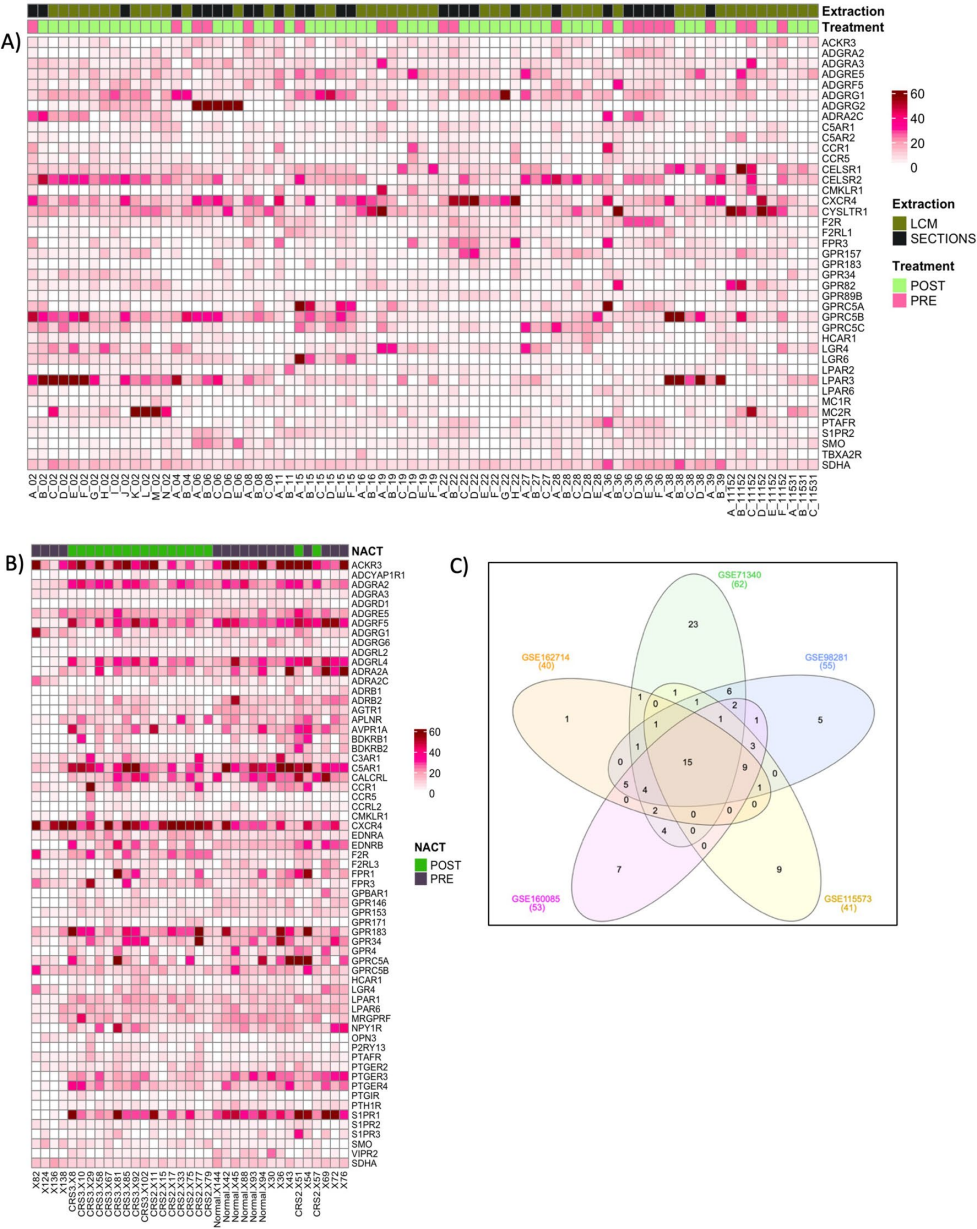
Heatmap of GPCR gene expression (RPKM) for **A**) GSE162714 and **B**) GSE98281. **C**) Venn diagram representing the numbers of expressed GPCR genes amongst the five datasets

Lab-to-lab variations in working protocols, including the total RNA used as starting material for RNA-seq experiments, can lead to differences in sequencing depth and distributions of gene read counts. To compensate for this, we introduced the expression level of the housekeeping gene *SDHA* as an internal gene expression standard. Amongst 15 housekeeping genes [32, 33], *SDHA* was selected based on its robustness, reflected by a low coefficient of variation (CV%) of 9.0% across the healthy tissue samples from data set GSE114493. Surprisingly, the most used control genes glyceraldehyde 3-phosphate dehydrogenase (*GAPDH*) and beta actin were much more variable with CV% values of 22.3% and 28.8%, respectively. This finding correlated with an earlier study by Bär et al. where *SDHA* was selected amongst ten other housekeeping genes as the best normalisation reference when comparing mRNA expression between independent tissue samples [32]. Expressed GPCRs across patient samples were systematically identified, defined as those with an expression level of at least 40% of *SDHA* expression, in at least 30% of samples within a data set. Figure 3 displays representative heatmaps of the two largest datasets showing expression levels of 40 and 62 genes (the remaining three datasets are shown in Figure S3).

#### Many GPCRs showed frequent but heterogeneous expression

All five datasets showed very distinct heterogeneous expression patterns for the selected GPCRs, with some receptors indicating links to known underlaying cancer related mechanisms such as migration, proliferation, invasion, angiogenesis, and metastasis [34]. For example C-X-C chemokine receptor type 4 (CXCR4) [35] and sphingosine-1-phosphate receptor 2 (S1PR2) [36] have been studied extensively in the past for their roles in cell migration, proliferation and invasion, and coagulation factor 2 thrombin receptor (F2R) is known to promote blood vessel development [37]. Overall, our heatmaps did not reveal obvious and consistent GPCR gene expression signatures across multiple ovarian cancer tissue samples in general or specific to disease sub-types. Whilst the expression for individual receptors at first sight appeared erratic across cancer samples, it was remarkable, that many of the selected receptors, nevertheless appeared at very significant frequencies. This may reflect the diverse genetic makeup of ovarian cancer tissues and could be due to a variation of underlaying mechanisms driving genomic instability in ovarian cancer cells. However, it is crucial to acknowledge that this heterogeneity could also arise from a multitude of other factors inherent to tumor biology including epigenetic modifications and the influence of the tumour microenvironment, which can strongly affect gene expression [38].

#### Several target receptors were found to be expressed across datasets

The variable GPCR expression profiles (Fig. 3A and B and S4) illustrated the heterogeneous landscape of ovarian cancer across various tumour subtypes, patients, and treatments. A Venn diagram (Fig. 3C) was generated to visualise GPCR expression in 151 cancer samples from 69 patients across the selected five datasets, leading to a ranking according to expression frequency. In summary, a total of 45 GPCRs were found to be consistently expressed in at least three or more datasets. Fifteen out of these 45 genes were expressed in all five datasets (Table 3), representing a subset of GPCRs with robust and recurrent expression patterns in ovarian cancer.

**Table 3:** GPCRs with frequent expression in at least three datasets*

**Five datasets**
*ACKR3*,* ADGRA2*,* ADGRA3*,* ADGRE5*,* ADGRG1*,* ADRA2C*,* C5AR1*,* CXCR4*,* F2R*,* FPR3*,* GPRC5A*,* GPRC5B*,* PTAFR*,* S1PR2*,* SMO*
**Four datasets**
*ADGRF5*,* ADGRG2*,* CCR1*,* CELSR1*,* CELSR2*,* CYSLTR1*,* GPR157*,* GPR183*,* GPRC5C*,* LGR4*,* LGR6*,* LPAR2*,* LPAR3*,* LPAR6*,* OPN3*
**Three datasets**
*ADGRL1*,* ADGRL2*,* C5AR2*,* CCR5*,* EDNRA*,* F2RL1*,* GPR34*,* GPR82*,* GPR89B*,* HCAR1*,* LGR5*,* MC1R*,* PTGIR*,* PTH2R*,* TBXA2R*

*All genes named according to the Human Genome Organization Gene Nomenclature Committee (HGNC, genenames.org), for non-abbreviated names see Table S2

### Final selection of potential target receptors

**Fig. 4:**
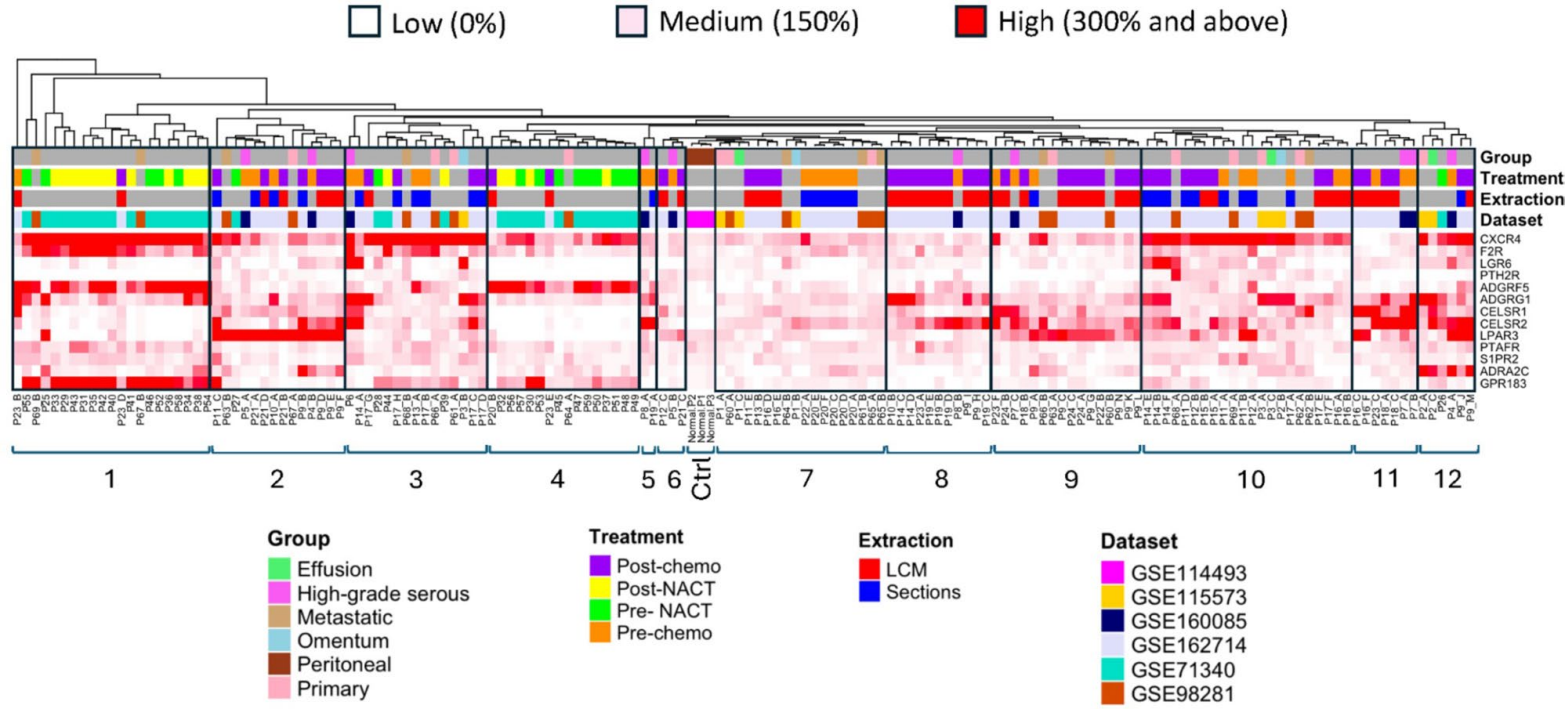
Expression pattern of 13 GPCRs across 151 ovarian cancer samples from 69 patients and three normal peritoneal samples from dataset GSE114493 were included as controls (Ctrl). Four different information sets are indicated on top of the heatmap: Group (Effusion, High-grade serous, Metastatic, Omentum, Peritoneal and Primary), Treatment (Post-chemo, Post-NACT, Pre-NACT and Pre-chemo), Extraction (laser capture microdissection (LCM) and sections) and Dataset (GSE114493, GSE115573, GSE160085, GSE162714, GSE71340 and GSE98281). Expression is represented on a scale of low (white, 0%), medium (pink, 150%) and high (red, 300%) with percentages relative to *SDHA*. Gray indicates when corresponding values were not available

Many tumours, especially high-grade serous ovarian carcinomas, originate from epithelial cells of the fallopian tubes and metastasise into the peritoneum during cancer progression [39]. Nanoparticles have been used in the past for intra-peritoneal drug delivery in murine tumour xenograft models for local treatment of ovarian cancer [40], and this route of administration is very likely to become more routinely used in the clinic, especially with novel nanomedicines arriving in the future [41]. For peritoneal administration of nanoparticles, it is important to develop a strategy to avoid exposure of healthy cells to the drug load within the peritoneum. For this purpose, filtering criteria were applied to select GPCRs with low expression levels in healthy peritoneal samples relative to cancerous ones. We focused particularly on the peritoneal healthy samples as comparators, since unintended adverse effects on these tissues were considered more detrimental than on fallopian tubes and ovaries, which are routinely removed during ovarian cancer surgery. The expression levels (RPKMs) of the 45 pre-selected GPCRs, across the 151 high-grade serous ovarian tumour samples, were systematically compared to healthy peritoneal samples. Only genes with minimal expression in cancerous tissues at least two standard deviations above the mean of healthy tissues, in at least 65% of the 151 samples (across 5 datasets) were retained. This arbitrary process led to the identification of 13 GPCRs that were regularly expressed across the cancer tissues at elevated levels in comparison to normal samples, thus reducing the likelihood of targeting healthy cells. In a next step, all RPKM expression data from 151 samples for the remaining 13 GPCRs were normalised against *SDHA* (%) and subjected to hierarchical clustering as shown in Fig. 4. The analysis allowed delineation of the entire patient tissue cohort into 12 clusters containing 2–22 samples each, plus the control group of three healthy peritoneal samples. Notably, clusters 1–4, 10 and 12 displayed a pronounced expression of *CXCR4*. *F2R* expression was predominantly elevated in clusters 1 and 3. *LPAR*3 was the dominant GPCR in clusters 2 and 9, with additional frequent expression in clusters 10–12. Four cell adhesion receptors (*ADGRF5*, *ADGRG1* and *CELSR1/2*) were observed to be frequently expressed at very high levels, except for clusters 6 and 7. Out of those, *ADGRF5* expression was markedly high in clusters 1 and 4. *GPR183* and *ADRA2C* were almost exclusively expressed in clusters 1 and 12, respectively. Finally, *LGR6*, *PTH2R*, *PTAFR*, and *S1PR2* exhibited low to medium expression levels in most clusters. Noteworthy, *LGR6* has also been extensively studied to develop melanoma targeting antibody drug conjugates [42], and it could be an emerging target for ovarian cancer treatment. Of particular interest was cluster 1, which demonstrated co-expression of *CXCR4*, *F2R*, *ADGRF5*, and *GPR183* in nearly all patient samples. Closer inspection revealed that the 21 samples from this cluster have been derived from 20 patients, which were part of three independent datasets. As shown in Fig. 4, the 151 patient samples did not segregate into distinct clusters based on dataset, extraction method, pre/post cancer treatment, and other groups based on cancer location or type. In summary, our findings revealed a group of GPCRs with frequent but rather random expression across ovarian cancer tissue samples, hence strongly supporting the need for a personalised approach, including analysis of receptor expression in tumour biopsies, followed by case-to-case preparation of suitable drug loaded nanomedicines, decorated with targeting ligands.

The encountered challenges for the design of functional nanoparticles will strongly depend on the chosen targeting ligand. Interestingly, the final 13 selected GPCRs could be assigned to four distinct groups according to their ligand types, namely: peptide-activated receptors (*CXCR4*, *F2R*, *LGR6* and *PTH2R*), lipid-activated receptors (*LPAR3*, *PTAFR* and *S1PR2*), cell adhesion receptors (*ADGRG1*, *ADGRF5*, *CELSR1* and *CELSR2*), and other small molecule-activated receptors (*ADRA2C* and *GPR183*). Peptide receptors are particularly amenable for targeting with ligand-decorated nanoparticles. Notably, CXCR4 with its well-characterized CXCL12 ligand protruding from the activated receptor can be harnessed to inspire the design of suitable targeting agonists and antagonists [43]. This peptide receptor is known to promote cancer cell migration and plays a role in the development of drug resistance, and radionuclide-based imaging and therapy are currently under investigation [44, 45]. Our data revealed moderate to high *CXCR4* expression in most ovarian cancer tissues examined in this study. Remarkably, whilst *CXCR4* and *F2R* were found to be expressed in 71 and 57% of all samples, respectively, they were also co-expressed in 44% of all samples, to suggest possible dual targeting via these receptors to further enhance the efficacy of future cancer treatments.

For adhesion receptors, the fluctuating expression of *ADGRF5*, *ADGRG1* and *CELSR1/2* across datasets underscored the potential for these receptors in precision medicine (Fig. 4). An endogenous peptide ligand has recently been discovered for ADGRG1 and might be relevant for targeting strategies with nanomedicines [46]. *CELSR1* and *CELSR2* were consistently expressed in at least four datasets, representing an emerging class of targets for precision medicines. Although ligands for most of these receptors are yet to be discovered, their frequent overexpression makes them good candidates for future research into active targeting. A previous study by Kubler et al. also demonstrated elevated expression of *CELSR2* in other cancer types, including prostate and breast [47]. Moreover, adhesion receptors are known to have large N-terminal extracellular domains [48] making them suitable for antibody-based targeting. The development of engineered antibodies could potentially exploit these receptors to mediate selective uptake of drug loaded nanoparticles.

Lipid-activated receptors represented by LPAR3, S1PR2 and PTAFR were included in our final selection which could open new avenues for innovative drug delivery approaches. The targeting of these receptors is expected to be challenging due to the hydrophobic nature of their ligands, which could compromise nanoparticle stability following chemical conjugation [49]. However, with rapidly advancing technologies, it might be possible to engineer nanoparticles that will present lipid-like structures or derivatives to engage with these targets, allowing specific delivery of therapeutic agents to ovarian cancer cells.

Finally, receptors with small molecule ligands like ADRA2C and GPR183 that had moderate to high expression in many samples could offer a unique platform, highlighting their potential as biomarkers and therapeutic targets. While attaching small molecules to nanoparticles presents challenges, such as ensuring stable conjugation and maintaining the biological activity of the molecule, advancements in ligation chemistry and nanoparticle design could pave the way for more precise and effective targeting of receptors.

### In vitro assessment of mRNA expression for selected target receptors using ovarian cancer patient-derived samples and cell lines

**Fig. 5:**
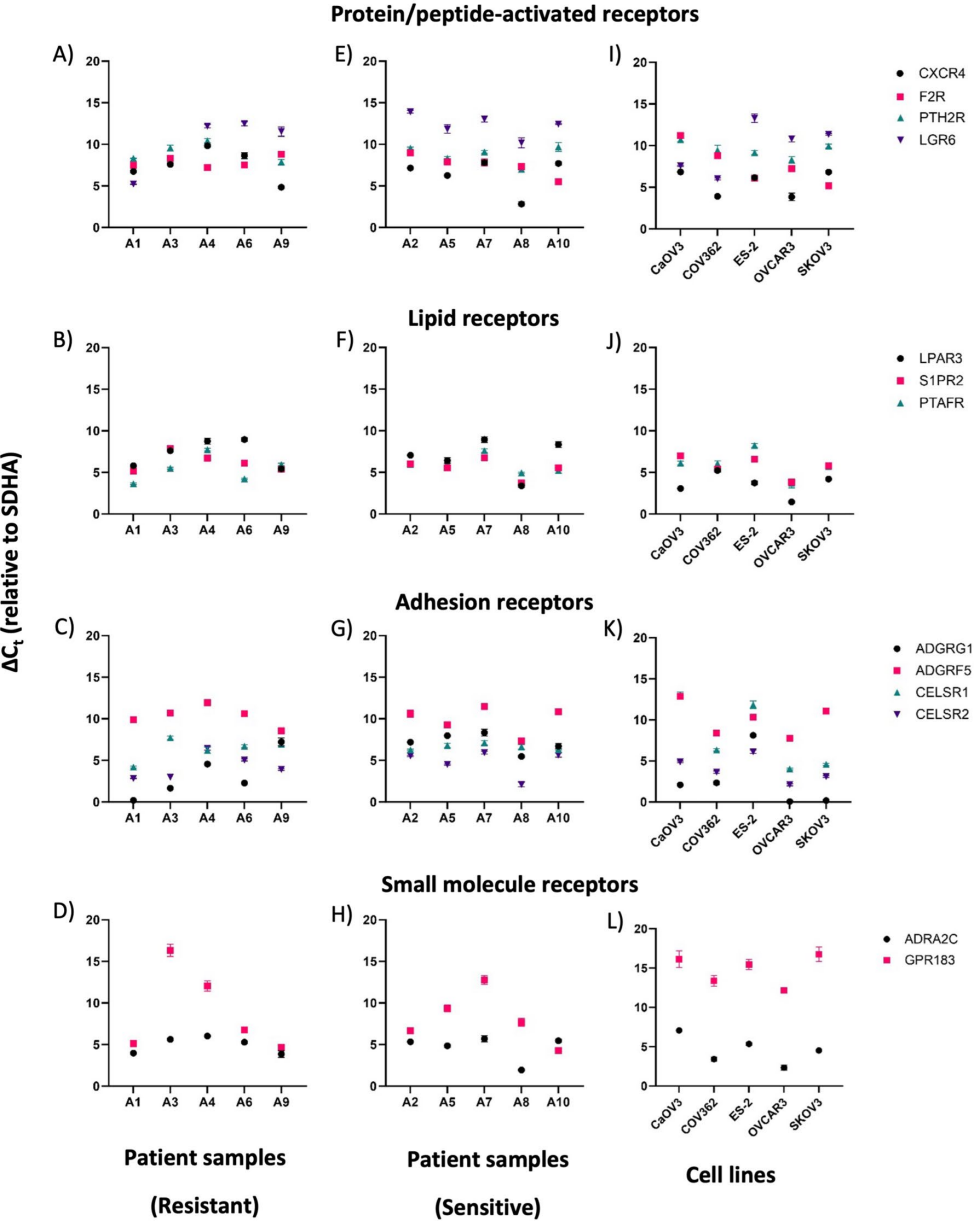
RT-qPCR experiments for 13 GPCRs. Panels A-D show results for chemotherapy resistant, and panels E-H chemotherapy sensitive patient-derived samples. Panels I-L depict data for established ovarian cancer cell lines. ΔC_t_ values are given relative to *SDHA* expression (individual experiments in triplicates)

To confirm the expression of GPCRs within ovarian cancer cells, our study was extended to in vitro analyses using patient-derived specimens. The RT-qPCR technique was used to confirm mRNA expression for the selected GPCRs in ten patient ascites samples (including chemotherapy resistant and sensitive samples), and the results demonstrated a consistent expression of the *SDHA* housekeeping gene across all samples, with a C_t_ value range of approximately 20–23 cycles (except patient sample A8, C_t_ = 25.66), underscoring the gene’s robustness as an internal control for comparative analyses. C_t_ values > 35 cycles were interpreted as mRNA copy numbers below limit of detection in the respective samples. ΔC_t_ values of all target GPCRs relative to the *SDHA* reference are depicted in Fig. 5 (A-H), revealing a large ΔC_t_ range of 0.19–16.33, indicating 1.14-82,379 times less expression relative to *SDHA*. In line with the preceding RNA-seq data analysis, the RT-qPCR results confirmed high heterogeneous expression levels with most ΔC_t_ values observed between 5 and 10, suggesting expression levels of approximately 32–1,000 times lower than *SDHA*.

Peptide activated GPCR genes, notably *CXCR4* and *F2R*, displayed sustained expression across a diverse array of patient samples (Fig. 5A and E), encompassing those from individuals with confirmed resistance to conventional chemotherapy. In contrast, *LGR6* displayed a very diverse expression pattern with ΔC_t_ values ranging from approximately 5 to 14, which was in line with the RNA-seq data shown in Fig. 4. Three out of the ten patient samples gave C_t_ values > 35, indicating lack of gene expression. However, it needs to be noted that the primer pair for *LGR6* detection was the only one with a primer efficiency significantly lower than 90% (Table S3), potentially leading to the relatively low C_t_ values. The selected lipid receptors (*LPAR3*, *S1PR2* and *PTAFR*) appeared consistent, showing expression in most patient samples. Furthermore, adhesion receptors such as *ADGRG1* and *CELSR2* also exhibited elevated expression in both resistant and sensitive patient samples. *ADRA2C* demonstrated high expression levels, while *GPR183* showed lower expression.

Patient-derived ascites samples were routinely checked to exclude contamination with non-cancerous cells, such as fibroblasts. However, it was not possible to fully exclude any contamination, and due to the nature of RT-qPCR, even relatively small contents of non-cancerous cells could affect the results. Therefore, additional testing was performed with five established ovarian cancer cell lines to assess expression in pure cancer cell cultures. The expression patterns observed in the cell lines, as depicted in Fig. 5 (I- L), were in line with patient sample data, apart from the *GPR183* gene, resulting in ΔC_t_ values between 12 and 17. Moreover, the C_t_ values for this gene was > 35 in all five cell lines evaluated, and we excluded this receptor from the target list.

Overall, the RT-qPCR experiments confirmed gene expression for all selected target receptors in patient-derived samples and established cell lines, except *GPR183*. Specifically, *CXCR4* and *F2R* stand out by displaying significant expression levels in PCR analyses, thereby confirming co-expression patterns observed with RNA-Seq data analysis (Fig. 4). The simultaneous expression of *CXCR4* and *F2R* further strengthen the potential for co-targeting strategies that could exploit synergistic effects to inhibit cancer progression and enhance treatment efficacy. To bolster the molecular evidence, flow cytometry experiments were conducted to prove the presence of the CXCR4 and F2R proteins in the cancer cells.

### Confirmation of protein expression for two peptide-activated receptors

Moderate correlation between mRNA expression and protein levels have been shown in the past [50], and therefore we used RNA-seq derived CPM data as initial proxies to identify GPCRs with relevant expression in ovarian cancer tissues, followed by confirmation of gene expression in patient-derived samples and established ovarian cancer cell lines. However, protein expression levels need to be tested to confirm sufficient expression prior to embarking on nanoparticle-based drug targeting projects. This needs to be done individually for all receptors, also in additional cancer probes, to further assess expression in cancer cells versus cancer associated cells of the tumour microenvironment. Since this was beyond the scope of the current study, we focussed on two peptide activated receptors, as these were deemed the most feasible to develop novel nanomedicines. The natural ligands for CXCR4 and F2R, or modified versions, are amenable to attach on nanoparticle surfaces. CXCR4 has been explored for specific targeting of various cancer types in the past [51–53], but it is novel in the context of ovarian cancer, while F2R is a newly suggested target, specifically for ovarian cancer.

**Fig. 6:**
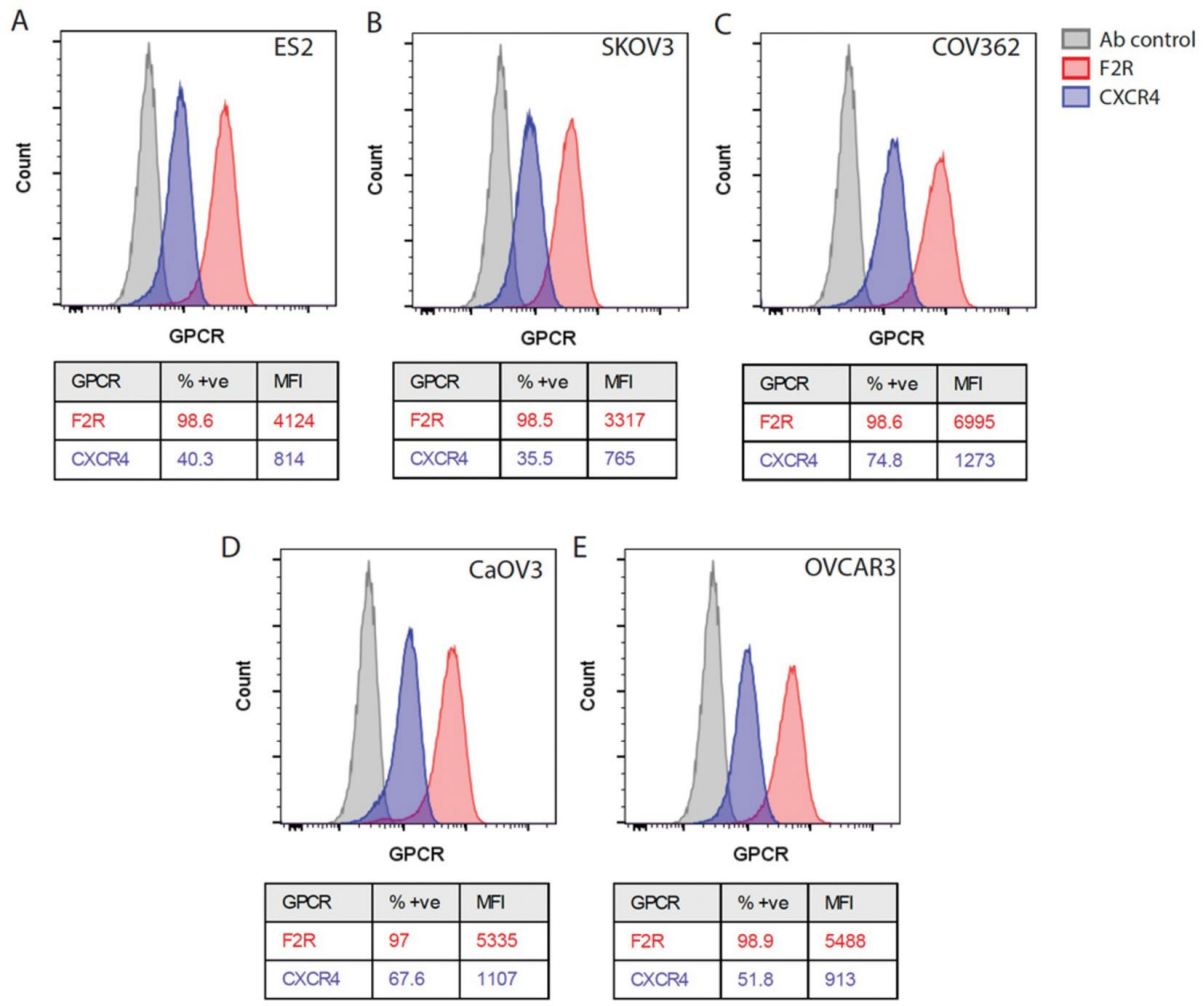
Flow cytometry analysis of CXCR4 and F2R expression in five ovarian cancer cell lines (A-E). The percentage of positive cells (%+ve) and the mean fluorescence intensity (MFI) are shown for both receptors in all cell lines

Figure 6 shows flow cytometry experiments performed with five cell lines using specific CXCR4 and F2R antibodies on cells which have been fixed and permeabilised. The detailed information on sample preparation and gating strategy is outlined in Figure S5. The percentage of positive cells (%+ve) and the mean fluorescence intensities (MFI) are tabulated for each receptor, indicating the proportion of cells expressing the receptor and the average expression level. F2R was ubiquitously expressed in all cell lines at very high percentages and high MFI values, suggesting a robust receptor expression in these cancer cell lines. CXCR4 showed variable expression, with lower percentages and MFI in some cell lines (notably SKOV3) compared to F2R, indicating a more heterogeneous expression of this receptor among cell lines.

Here we conclude that both receptors showed robust protein expression in all five ovarian cancer cell lines, indicating them as suitable targets for the design of future nanomedicines. The natural peptide ligands, or fragments thereof, will be the obvious choice to target the receptors when running in vitro feasibility studies on cultured cells. For later in vivo applications, it might be necessary to develop modified ligands to overcome instability issues inherent to peptide ligands.

## Conclusions and future directions

By integrating RNA-seq data analysis with in vitro validation, we have identified a selective group of GPCRs with promising application in targeted therapies. These receptors have demonstrated moderate to high expression in ovarian cancer tissues, suggesting their suitability for the development of personalised treatments that aim to deliver anti-cancer agents directly to tumour cells while avoiding healthy tissues.

Each of these targets will need further in-depth validation to study protein expression in a broad range of ovarian tumour subtypes, including different grades and stages. More detailed exploration of the identified GPCRs in a broader range of ovarian cancer subtypes will be pivotal in understanding the spectrum of their expression and activity. In addition to this, detailed structure and function analysis will be necessary to design suitable targeting ligands for the preparation of experimental nanomedicines. In vivo animal models can be employed to assess the therapeutic impact of GPCR-targeted nanomedicines. Another avenue for future research involves the investigation of the molecular pathways associated with these GPCRs and the effect of cell cycle on their expression, this could reveal novel mechanisms of tumour progression and potential resistance to therapy. Lastly, considering the genetic diversity among ovarian cancer patients, efforts should also be focused on establishing predictive biomarkers that can identify individuals who would benefit the most from GPCR-targeted treatments, thereby advancing the personalised medicine approach in oncology.

### Electronic supplementary material

Below is the link to the electronic supplementary material.

Supplementary Material 1

## Data Availability

No datasets were generated or analysed during the current study.

## Declarations

### Data accessibility

The datasets used and/or analysed during the current study are available from the corresponding author on reasonable request.
