# Supplementary material for: Unveiling G-protein coupled receptors as potential targets for ovarian cancer nanomedicines: from RNA sequencing data analysis to in vitro validation
Source: J Ovarian Res. 2024 Jul 27;17:156. doi: 10.1186/s13048-024-01479-0 (PMC11282829; doi:10.1186/s13048-024-01479-0)
Supplement: Supplementary file 1 — Supplementary Material 1 [file 13048_2024_1479_MOESM1_ESM.docx]

**Supplementary material**

**Figure S1.** Boxplot representing RNA-seq data of 20 samples from the GSE98281 dataset before (top) and after (bottom) TMM normalisation.


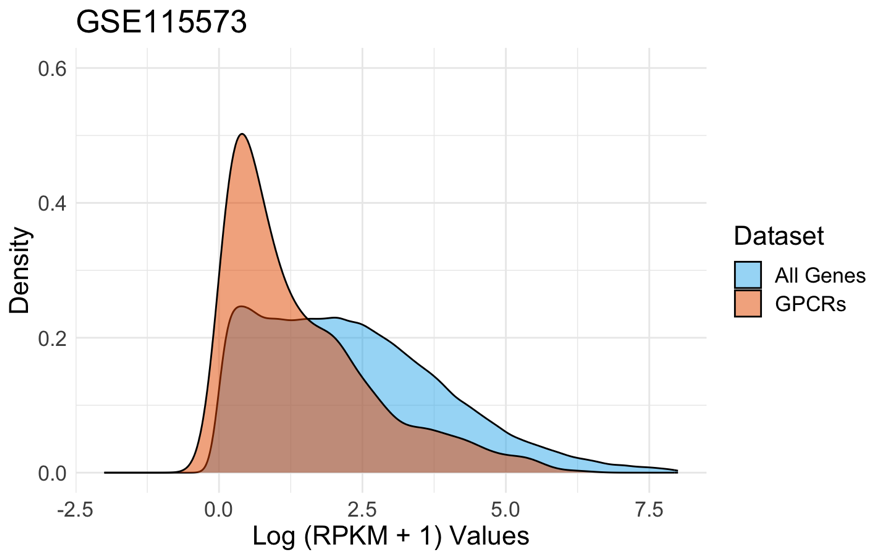

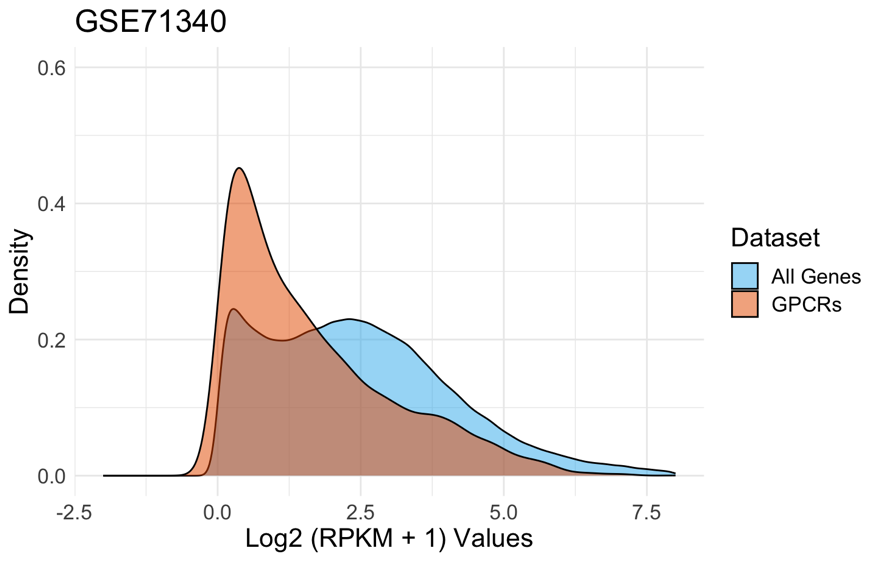

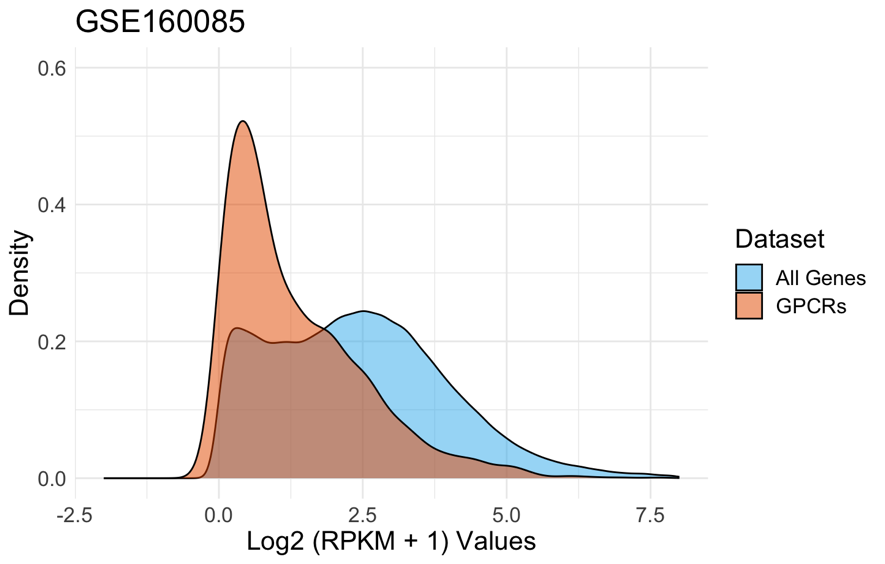

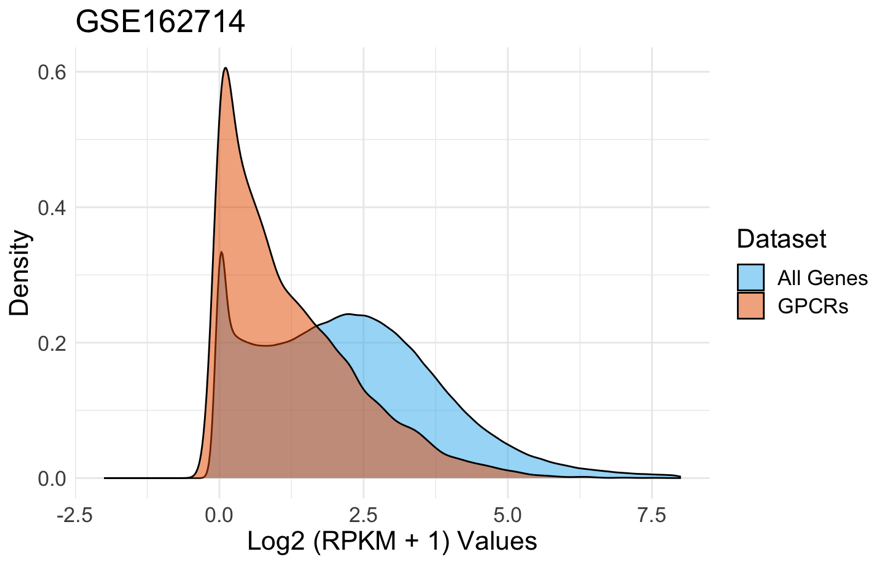


**Figure S2.** Density plot representing the log2 (RPKM+1) values of four datasets (GSE162714, GSE160085, GSE15573 and GSE71340) with blue and orange colour representing all genes and GPCRs, respectively.


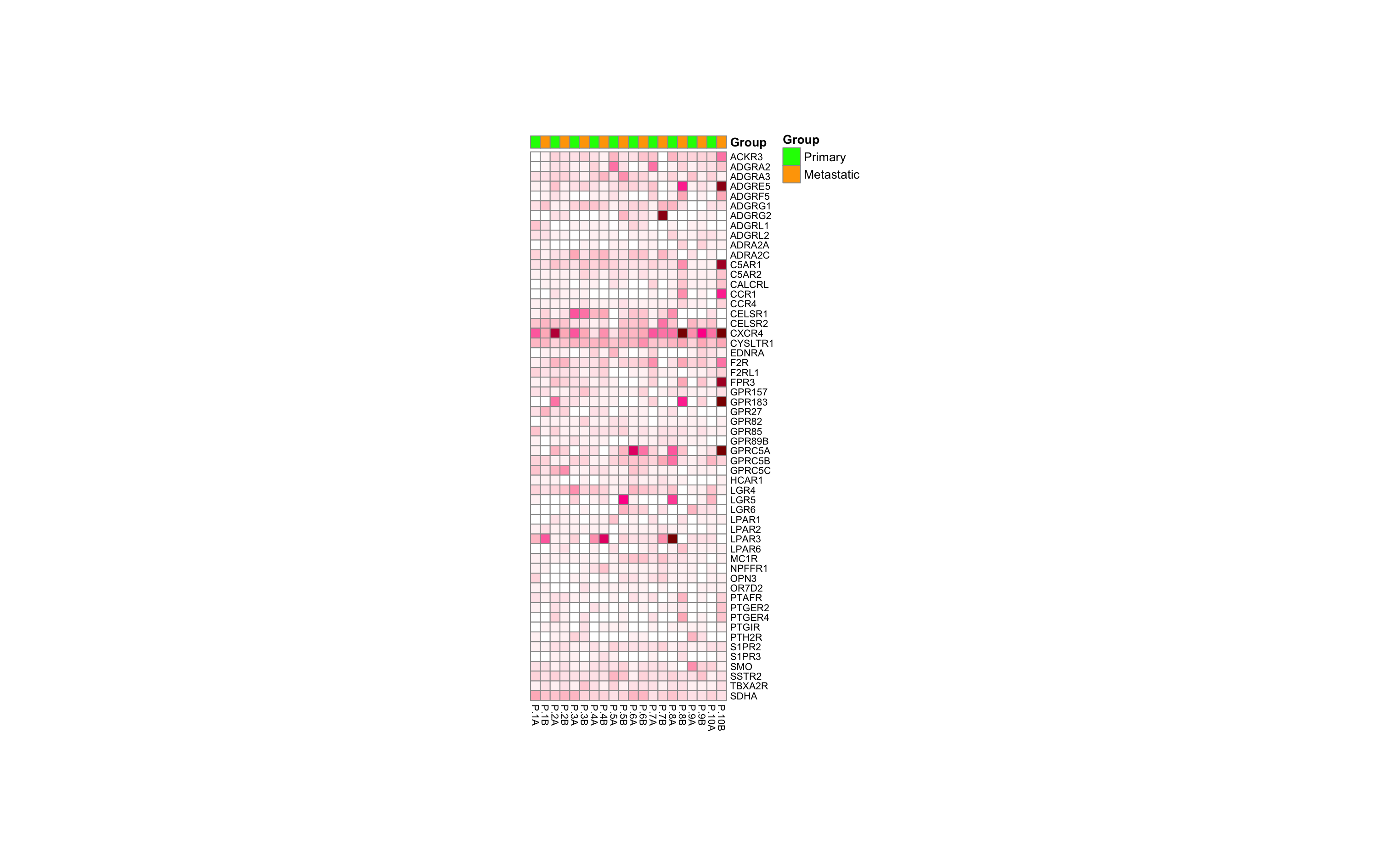

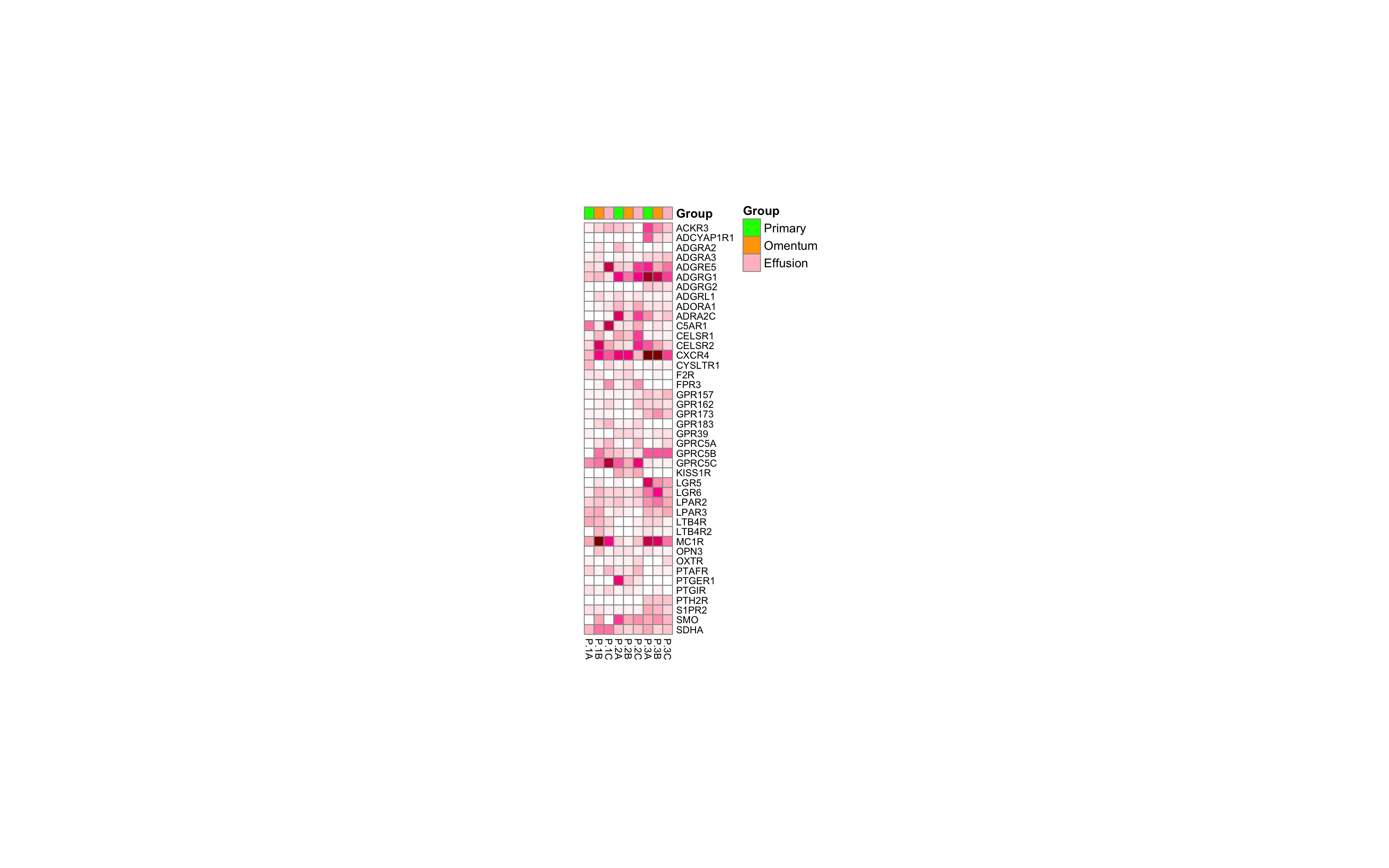

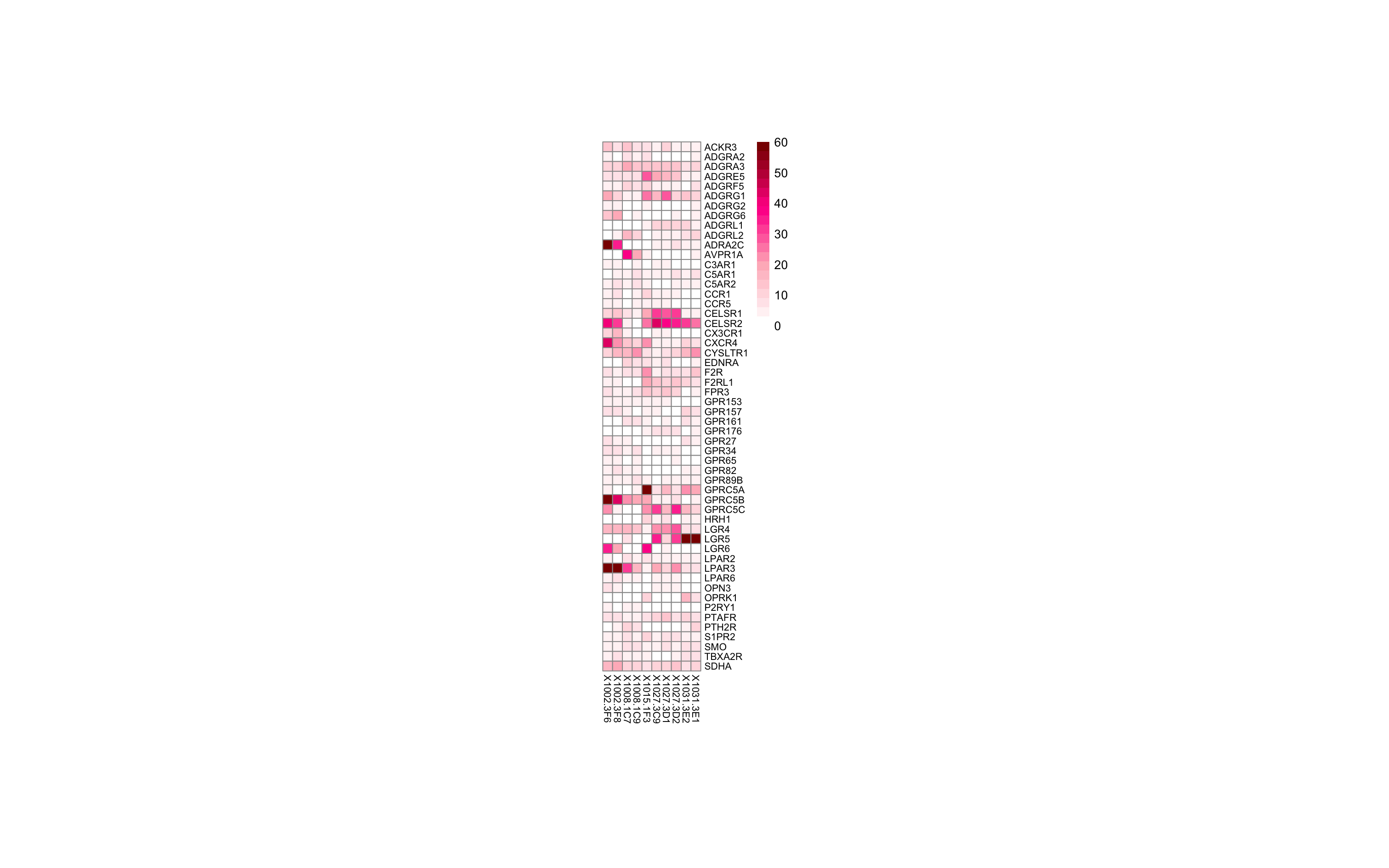


A)

B)

C)

**Figure S3.** Heatmaps representing expression of selected GPCR genes in datasets A) GSE98281, B) GSE115573 and C) GSE160085. Indicated groups: green and orange colour in GSE98281 indicate primary and metastatic samples, respectively; green, orange, and pink in GSE115573 indicate primary, omentum and effusion samples.

**Table S1.** Nucleotide sequences of RT-qPCR primers. Primers were designed using the NCBI primer design tool.

| Receptor name | Forward primer | Reverse primer |
| --- | --- | --- |
| SDHA | CTACGACACCGTGAAGGGCT | TGTCCACCAAATGCACGCTG |
| ADGRG1 | TGGTGGACTTCAGCAGCCAA | TGCAGAGTCACATTCTTCGGC |
| LPAR3 | TCCAACCTCATGGCCTTCCTC | GGTCCAGCATACCACAAACGC |
| F2R | AGAGAGGGTGAAGCGGAGCA | TGACCGGGGATCTAAGGTGG |
| CXCR4 | GGGCAGAGGAGTTAGCCAAG | GTAGTGGGCTAAGGGCACAAGA |
| ADRA2C | ACTGGTACTTCGGGCAGGTG | GGCTGATGGCACACAGATGC |
| LGR6 | CTGGACCCCCTGACGGCTTA | CAGCTCCTCCAAGAAGCGCA |
| ADGRF5 | CCTCGAGGTTCACCTCACCTG | TCCAGTTCAGTGCAGCTTTGG |
| GPR183 | AGACCCGAACGAGTCACTGAT | AGTTGCAGAGGGCGGAGTAA |
| S1PR2 | TGGAAACGCAGGAGACGACCTC | CGAGTGGAACTTGCTGTTTCGG |
| PTAFR | AAGCCGTCCAGGAAACATGC | CTTCAGCTGCAGTGACCGTG |
| CELSR1 | CGGGCCGTACTGTGAGAACA | GTCCTGGGCTAGGAGCTTGT |
| CELSR2 | GTAGCCTGCCTGACCCTTGT | TGCGGGTACACACAGACTGG |
| PTH2R | ACGTCTGGGGTTGGCTAATGC | GGGCCAACAAATGAGTCCATCC |

**Table S2.** Abbreviated form of all GPCR genes

| Gene | Gene name |
| --- | --- |
| *ACKR3* | Atypical chemokine receptor |
| *ADGRA2* | Adhesion G protein-coupled receptor A2 |
| *ADGRA3* | Adhesion G protein-coupled receptor A3 |
| *ADGRE5* | Adhesion G protein-coupled receptor E5 |
| *ADGRG1* | Adhesion G protein-coupled receptor G1 |
| *ADRA2C* | Adrenoreceptor alpha 2C |
| *C5AR1* | Complement C5a receptor 1 |
| *CXCR4* | C-X-C motif chemokine receptor 4 |
| *F2R* | Coagulation factor II thrombin receptor |
| *FPR3* | Formyl peptide receptor 3 |
| *GPRC5A* | G protein-coupled receptor class C group 5 member A |
| *GPRC5B* | G protein-coupled receptor class C group 5 member B |
| *PTAFR* | Platelet activating factor receptor |
| *S1PR2* | Sphingosine-1-phosphate receptor 2 |
| *SMO* | Smoothened, frizzled class receptor |
| *ADGRF5* | Adhesion G protein-coupled receptor F5 |
| *ADGRG2* | Adhesion G protein-coupled receptor G2 |
| *CCR1* | C-C motif chemokine receptor 1 |
| *CELSR1* | Cadherin EGF LAG seven-pass G-type receptor 1 |
| *CELSR2* | Cadherin EGF LAG seven-pass G-type receptor 2 |
| *CYSLTR1* | Cysteinyl leukotriene receptor 1 |
| *GPR157* | G protein-coupled receptor 157 |
| *GPR183* | G protein-coupled receptor 183 |
| *GPRC5C* | G protein-coupled receptor class C group 5 member C |
| *LGR4* | Leucine rich repeat containing G protein-coupled receptor 4 |
| *LGR6* | Leucine rich repeat containing G protein-coupled receptor 6 |
| *LPAR2* | Lysophosphatidic acid receptor 2 |
| *LPAR3* | Lysophosphatidic acid receptor 3 |
| *LPAR6* | Lysophosphatidic acid receptor 6 |
| *OPN3* | Opsin 3 |
| *ADGRL1* | Adhesion G protein-coupled receptor L1 |
| *ADGRL2* | Adhesion G protein-coupled receptor L2 |
| *C5AR2* | Complement C5a receptor 2 |
| *CCR5* | C-C motif chemokine receptor 5 |
| *EDNRA* | Endothelin receptor type A |
| *F2RL1* | F2R like trypsin receptor 1 |
| *GPR34* | G protein-coupled receptor 34 |
| *GPR82* | G protein-coupled receptor 82 |
| *GPR89B* | G protein-coupled receptor 89B |
| *HCAR1* | Hydroxycarboxylic acid receptor 1 |
| *LGR5* | Leucine rich repeat containing G protein-coupled receptor 5 |
| *MC1R* | Melanocortin 1 receptor |
| *PTGIR* | Prostaglandin I2 receptor |
| *PTH2R* | Parathyroid hormone 2 receptor |
| *TBXA2R* | Thromboxane A2 receptor |

**Primer efficiency**

**Figure S4**. Standard plot representing the template quantity versus the cycle threshold observed for SDHA (n = 2).

The following mathematical equation was used to calculate the primer efficiency:

$$Efficiency\left( \% \right)={(10}^{\frac{-1}{Slope}}-1)*100$$

**Table S3**. Primer efficiency (%) for all the primer pairs:

|  | Average cycle threshold (C_t_) for cDNA stock | | | |  |  |
| --- | --- | --- | --- | --- | --- | --- |
| Genes | **1/10** | **1/100** | **1/1000** | **1/10000** | **R^2^** | **Primer efficiency (%)** |
| *SDHA* | 22.36 | 25.36 | 28.28 | 32.59 | 0.991 | 98.39 |
| *ADGRG1* | 22.19 | 25.17 | 28.28 | 32.51 | 0.993 | 96.55 |
| *ADGRF5* | 29.45 | 32.34 | 34.80 | 40.45 | 0.961 | 91.48 |
| *CELSR1* | 26.05 | 29.13 | 32.19 | 37.21 | 0.983 | 87.81 |
| *CELSR2* | 24.47 | 27.16 | 30.37 | 33.85 | 0.997 | 108.54 |
| *LPAR3* | 23.30 | 26.31 | 29.39 | 32.87 | 0.999 | 106.31 |
| *S1PR2* | 26.37 | 29.71 | 32.50 | 37.00 | 0.990 | 94.23 |
| *PTAFR* | 25.46 | 28.84 | 32.36 | 36.08 | 1.000 | 91.66 |
| *CXCR4* | 26.32 | 29.24 | 32.53 | 36.84 | 0.992 | 93.56 |
| *F2R* | 29.35 | 31.46 | 34.21 | 39.62 | 0.950 | 98.62 |
| *PTH2R* | 29.87 | 32.92 | 37.16 | 38.76 | 0.974 | 110.60 |
| *ADRA2C* | 24.69 | 27.77 | 31.13 | 35.13 | 0.996 | 94.17 |
| *LGR6* | 31.11 | 34.73 | 39.41 | 43.72 | 0.998 | 71.85 |
| *GPR183* | 35.41 | 39.59 | 41.21 | 45.68 | 0.973 | 91.15 |


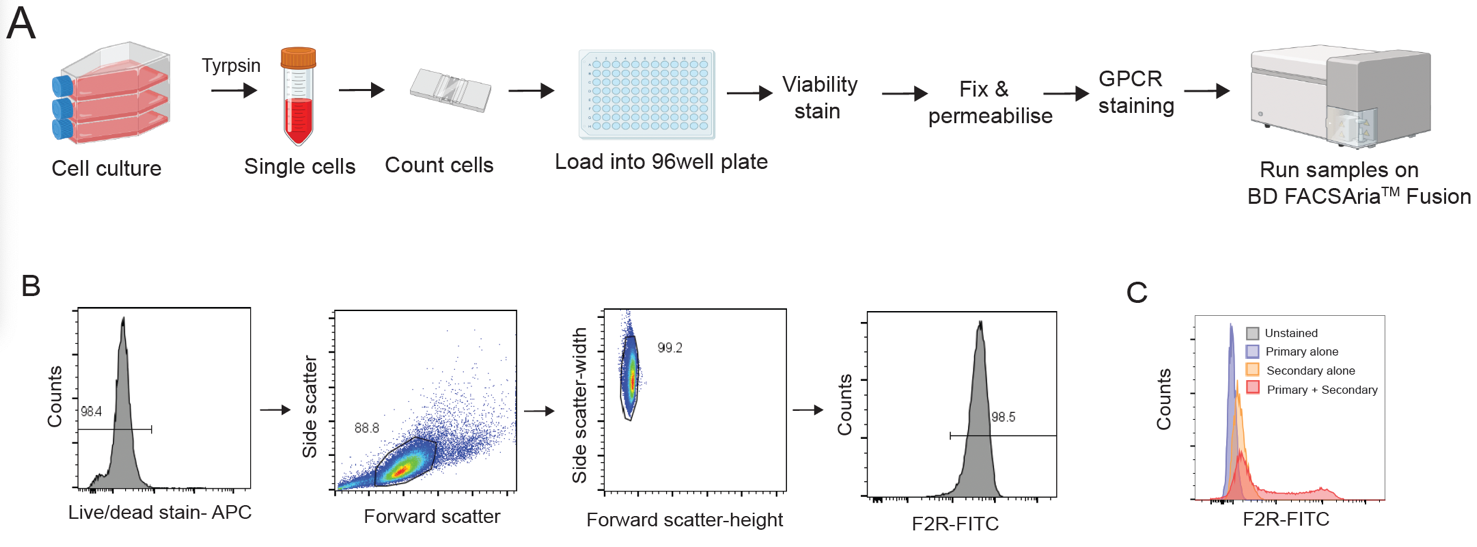


**Figure S5**. Schematic representation of sample preparation for flow cytometry and gating strategy: A) Steps involved from cell culture to cell counting, staining, fixing and permeabilising before flow cytometric analysis; B) gating strategy to quantify cell counts; C) four individual peaks representing unstained cells, primary or secondary antibody stained cells as controls, and primary plus secondary antibody stain for detection of F2R. Parts of the figure were created from BioRender.com.
